# Factors Associated With the Use of Radiation Therapy in Patients With Stage III Non-small Cell Lung Cancer in Alberta, Canada: A Population-based Study

**DOI:** 10.7759/cureus.851

**Published:** 2016-10-27

**Authors:** Hong-wei Liu, Marc Kerba, Gerald Lim, Zsolt Gabos, Ivo A Olivotto, Anil Abraham Joy, Wilson Roa, Zoann Nugent, Harold Lau

**Affiliations:** 1 Radiation Oncology, Central Alberta Cancer Center; 2 Department of Oncology, Tom Baker Cancer Centre, Calgary; 3 Radiation Oncology, Cross Cancer Center, University of Alberta; 4 Oncology, Cross Cancer Institute, University of Alberta; 5 Department of Epidemiology and Cancer Registry, Cancer Care Manitoba, University of Manitoba, Canada; 6 Radiation Oncology, Tom Baker Cancer Centre, Calgary

**Keywords:** stage iii non-small cell lung cancer, overall survival, resource of health facility, practice pattern

## Abstract

**Background:**

Cancer care in Alberta, Canada is publicly funded and provides patients with access to health care facilities and providers. The distribution of patients and health services across Alberta presents challenges to the delivery of cancer care, especially radiation therapy. In this study, we examined the association between patient and health system factors, the use of radiation therapy and survival outcomes in patients with stage III non-small cell lung cancer (NSCLC).

**Patients and methods:**

The provincial cancer registry was used to identify all patients who presented with clinical stage III NSCLC, diagnosed from 2005 to 2007, in Alberta. Patient characteristics, diagnostic method, treatment modality and treatment outcomes were collected from provincial health information systems for analyses. Factors influencing overall survival (OS) were analyzed using Cox proportional hazards models.

**Results:**

Nine hundred twenty-nine patients were identified. Sixty-two percent of patients received radiation therapy (RT) as part of their initial cancer treatment and had a median OS of 1.04 vs. 0.34 years with a hazard ratio (HR) of 0.54. On multivariable analysis, patients who were less likely to receive any therapy were older, had higher comorbidity scores and were registered in community cancer centers without radiation therapy infrastructure. Patients registered in tertiary cancer centers had a higher likelihood of accessing multimodality treatment than patients in community centers, with a statistical significance of P<0.001 after correcting for age, gender, histology, substage, and comorbidity.

**Interpretation:**

Improving access to radiotherapy treatment for patients presenting to non-radiation therapy centers at diagnosis has the potential to decrease variations in cancer care and improve cancer control outcomes in clinical stage III NSCLC.

## Introduction

Lung cancer is a leading cause of cancer-related mortality in Canada [1]. NSCLC accounts for 80% of lung cancer; about 30% of NSCLC presents with locally advanced disease at clinical stage III. The survival of clinical stage III NSCLC patients is poor and most patients are not eligible for surgical resection. Despite established, evidence-based guidelines, the management and outcomes of clinical stage III NSCLC continue to vary significantly at a national and international level [2-5]. The usual treatment paradigm is radical radiation therapy (RT) with or without concurrent chemotherapy [6].

In Alberta, Canada, many patients with a clinical cancer diagnosis, including stage III NSCLC, reside at a distance of more than 100 km from the tertiary cancer center, a challenge to the provision of reasonable access to cancer care. It has been observed that challenges such as geographical distance from a treating cancer center is associated with a decreased likelihood of receiving active treatment [7]. The impact of this unmet need in care on outcomes in lung cancer, exacerbated by the distribution of cancer services, is unknown.

Patients with clinical stage III NSCLC may not receive active management due to a variety of health system factors. In addition to oncologist's judgment, multimodality practice pattern, referring physician awareness of guidelines and patient beliefs or socioeconomic factors are known to influence cancer management and outcomes [4,8-11]. The current study was undertaken to review patient, disease and health system factors in clinical stage III NSCLC management that might influence treatment decision patterns [4,11-12] and survival outcomes, with the aim to inform efforts that may then minimize disparities in cancer care [5].

The University of Calgary granted IRB approval for this study and the approval number is E-27173. Informed consent was obtained from the patients for this study.

## Materials and methods

### Data source

This retrospective population-based study utilized a cohort design and examined patients diagnosed in Alberta, Canada, the fourth largest province with a population of more than four million inhabitants. Between 2005 and 2007 Alberta had two academic/tertiary cancer centers with RT capability and four major community cancer centers without RT. Study data was extracted from the provincial cancer registry following provincial research ethics board approval.

All Alberta residents presenting with clinical stage III NSCLC diagnosed between January 1, 2005, and December 31, 2007, were identified. Staging was determined using the American Joint Committee on Cancer, sixth edition. Oncologic management was extracted from the provincial cancer registry. Initial treatment was defined as "treatment planned and administered to the primary cancer site within six months of diagnosis." Patients who received no initial treatment were identified with the codes of “none,” “refused,” or “observation.” Patient factors including age, sex, collaborative stage, histology confirmation, initial registered cancer center and management information were collected. For patients who received initial active treatment including RT and chemotherapy, a review of the electronic medical chart was performed. RT delivery date, RT dose, fractionation and use of chemotherapy (either concurrent or sequential) as well as potential determinants including performance status, weight loss, lymph node sampling and positron emission tomography/computed tomography (PET/CT) staging were recorded.

The study patient cohort was linked to provincial health administration databases. Aggregated Clinical Risk Grouping (ACRG), a classification system for risk adjustment that assigns individuals one year prior to the cancer diagnoses, was used as a proxy measure of the impact of comorbidity [13-14]. ACRG-3 scores were collapsed into four categories of increasing comorbidity: 10-19=1, 20-49=2, 50-69=3, 70-99=4 for risk outcome analysis using Clinical Risk Grouping Software, V1.11 (3M, Murray, UT) [14].

### Statistical analysis

OS was defined from the date of diagnosis to date of death. The patients were censored on December 31, 2011. All statistical analyses were conducted using SAS software, V9.4 (SAS Institute, Inc., Cary, NC). The patient characteristics were compared using chi-square, Wilcoxon, and Kruskal-Wallis tests. OS was calculated using the Kaplan-Meier method and log-rank test. The association of study factors on cancer outcomes was tested using univariate and multivariate analyses with Cox proportional hazards modeling OS.

## Results

### Demographics of patients

A total of 929 patients were identified through the cancer registry. Eight hundred eleven patients (87.3%) initially were registered in the two large academic tertiary centers and 12.7% were registered in four community cancer centers (without RT capacity). Cancer center enrollment information including age, gender, histology, substage, comorbidity and variable patients’ management are listed in Table 1.

**Table 1:** General demographics and treatment details in study population *N=927: Two cases were unavailable. NOS: Not otherwise specified. RT: Radiation therapy.

	Tertiary Center A	Tertiary Center B	Community	P	Test
	N=332	N=479	N=118		
Age (median)	72	72	75	0.056	Kruskal-Wallis
Interquartile range	65-79	62-80	67-81		
Gender (%)				0.96	Chi-Square
Male/Female	58/42	58/42	59/41		
Histology (%)				0.47	Chi-Square
Adenocarcinoma	25	26	29		
Squamous cell	35	29	34		
NSCLC (NOS)	19	22	14		
Others	14	17	16		
No histology	7	6	8		
Subgroup stage (%)				0.16	Chi-Square
IIIA	29	23	28		
IIIB	71	77	72		
ACRG3 Score group (%)*				0.063	Chi-Square
<20	27	32	34		
20-39	20	17	11		
40-59	35	27	34		
60-99	19	23	21		
Lymph node sampling (%)	25.3	9.2	5.1	<0.0001	Chi-Square
PET/CT usage (%)	25	40	22	0.0003	Chi-Square
RT usage (%)	67	59	43	<0.0001	Chi-Square
Concurrent chemo-RT	26	14	9	<0.0001	Chi-Square

Sixty-two percent of patients had been assessed by an oncologist and received either initial palliative RT or more advanced RT included with radical management. The study found that 37.7% did not receive any initial active treatment. Patients receiving active therapy were of a younger age, had lower comorbidity scores and were more likely to be registered in tertiary centers. Table 2 details this patient cohort.

**Table 2:** Confounding factors between patients who had active treatment vs. no treatment

	Treated (N=579)	Untreated (N=350)	P	Test
Age (median)	69	78	<0.0001	Wilcoxon
Interquartile range	60-77	70-84		
Gender (%)			0.22	Fisher’s Exact
Male/Female	60/40	55/45		
Cancer center enrollment (%)			<0.0001	Chi Square
Tertiary center A	40	29		
Tertiary center B	51	52		
Community centers	9	19		
ACRG 3 score group (%)			0.0051	Mantal-Haenszel
<20	32	29		Chi-Square
20-39	19	13		
40-59	30	32		
60-99	19	26		

### Survival outcomes and the influence of study factors

**Figure 1:**
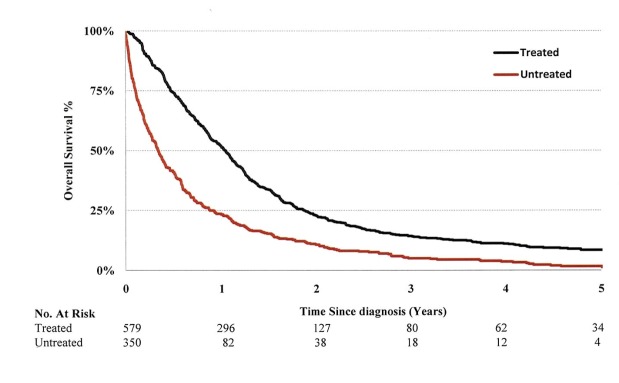
Kaplan-Meier overall survival curves for treated and untreated patients

Compared to patients receiving no therapy, patients assessed by an oncologist and receiving cancer treatment had better outcomes with a median OS of 12.4 months vs. 4.0 months and a statistical significance of P<0.0001 (Figure 1). After adjusting OS for variables of interest including pre-existing comorbidity (ACRG-3), patients with younger age, stage IIIA and active treatment had better OS (Table 3).

**Table 3:** Univariate and multivariate analyses predicting overall survival among whole study population HR: Hazard ratio. CI: Confidence interval.

	Univariate			Multivariate		
Factors	P	HR	95%CI	P	HR	95%CI
Age (per year)	<0.0001	1.02	1.01-1.03	0.04	1.01	1.001-1.01
Male vs. Female	0.041	1.15	1.01-1.32	0.25	1.08	0.94-1.25
Stage IIIA vs. IIIB	<0.0001	0.65	0.56-0.76	<0.0001	0.66	0.56-0.77
Treated vs. No	<0.0001	0.50	0.43-0.57	<0.0001	0.54	0.46-0.63
Cancer center enrollment						
Tertiary center B	ref					
Tertiary center A	0.079	0.88	0.76-1.02			
Community centers	0.12	1.18	0.96-1.45			
Community centers vs. tertiary’s	0.0322	1.24	1.02-1.52	0.2602	1.12	0.92-1.38
ACRG score group						
0-39 vs. 40-99	0.075	0.89	0.77-1.01	0.6225	0.97	0.84-1.11

**Figure 2:**
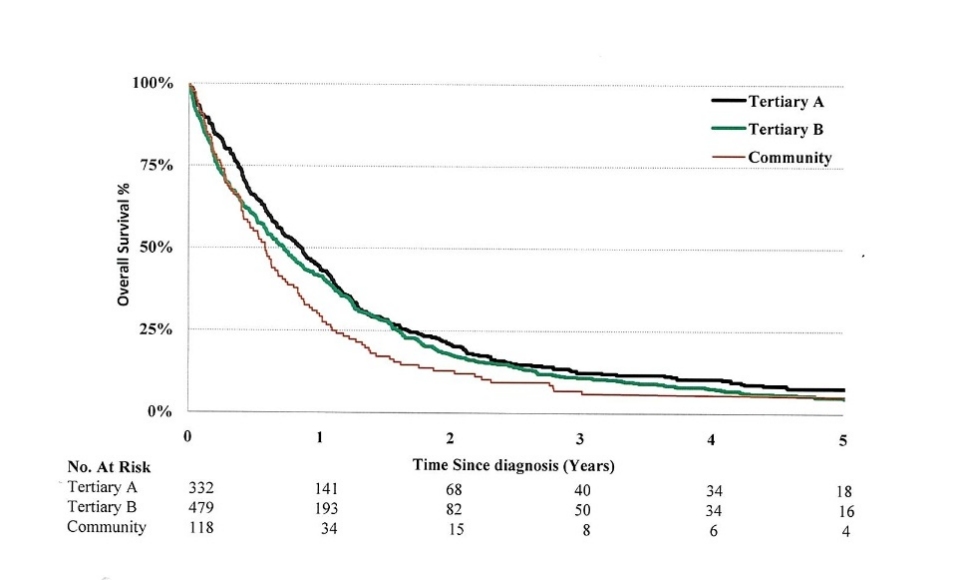
Kaplan-Meier survival curves for patients with three different enrollments

The patients presenting to community centers had inferior OS compared to those in tertiary centers, with a median OS of 9.1 months vs. 6.9 months (HR=1.24, 95% CI: 1.02-1.52, Figure 2).

For patients undergoing active initial treatment, Cox regulation analysis indicated patients with female gender, good performance, no weight loss, PET/CT staging and those who received radical RT or chemoradiotherapy had the best outcomes. For patients on treatment: age, ACRG3 score, and cancer center registration were not determinants for OS (Table 4).

**Table 4:** Multivariate analysis predicting overall survival among patients who had active treatment HR: Hazard ratio. CI: Confidence interval, RT: Radiation therapy. ECOG: Eastern Cooperative Oncology Group performance status score.

	P	HR	95% CI
Age (per year)	0.81	1.00	0.99-1.01
Male vs. Female	0.003	1.36	1.11-1.66
Stage III A vs. III B	0.3	0.89	0.71-1.11
Community vs. Tertiary center	0.85	0.97	0.68-1.37
Concurrent chemo-RT vs. RT	0.002	0.64	0.48-0.86
RT dose( >30Gy vs. <= 30Gy)	0.001	0.65	0.50-0.84
ACRG3 score group (0-39 vs. 40-99)	0.086	1.00	0.99-1.00
ECOG (0-1 vs. 2-4)	0.001	0.69	0.55-0.86
Weight loss (Yes vs. No)	0.021	1.29	1.04-1.59
PET/CT (Yes vs. No)	0.005	0.74	0.59-0.91
LN sampling (Yes vs. No)	0.93	1.01	0.79-1.30

## Discussion

In Alberta, Canada, the challenge of improving access to cancer care is compounded by the dispersed population and geographic distance [7]. Not surprisingly, the observed rate of RT utilization is consistent with studies previously reported [8,15-16]. The observed rate of RT treatment in this patient population (62.3%) is also lower than evidence-based estimates of the appropriate rate (~80% +/- 10% in stage III NSCLC), supporting that there is an unmet need for RT treatment in Alberta [4,11-12].

The utilization rate of RT for patients with NSCLC could vary for both medical and nonmedical reasons. Our findings suggest that patients registered in a community or rural setting have lower RT rates after accounting for known and measurable factors. Possible explanations include inadequate access to treatment due to barriers posed by geography or low referral rates (based on physician or patient preference) to the academic centers with radiation therapy capacity. The observed rural rate is consistent with other series [7]. Prevailing wait times for treatment were not an important influence on RT for lung cancer in Alberta between 2005 and 2007.

Improving quality of cancer care in the rural regions relies on providing comprehensive multimodality treatment options to all patients living in those regions. The Alberta Radiation Therapy Corridor, a project that has recruited specialized oncologists and built RT infrastructure in two community centers, was launched with this goal in mind. In this study, patients registered to community cancer centers all resided in rural areas. During the study period there were only two tertiary cancer centers located inside the two large cities in the province. Community cancer centers did not have the capability to provide comprehensive, RT-included multimodality therapy to meet patients’ needs, and access to telehealth-facilitated rounds was lacking. Our results demonstrate that a higher proportion of patients living in remote communities did not receive RT as part of their initial cancer treatment and appeared to have an inferior outcome compared to patients that had active therapy (Figure 1), even after adjusting for age, sex, histology, subgroup stage and comorbidity at enrollment.

Once outcome data mature following the implementation of the Alberta Radiation Therapy Corridor project, an examination of changes in utilization, access to RT and OS will be conducted.

Increasing patient age and comorbidity are known to be inversely associated with the likelihood of physician’s decision-making in recommending initial active treatment for patients with NSCLC [17-18]. Our results validate the finding that older patients or those with increased comorbidities are less likely to receive active treatment (Table 2). That said, prospective series examining outcomes in treating elderly patients with stage III NSCLC with curative intent achieved five-year survival rates similar to a younger population [17,19-21]. In our study, among patients who had active treatment, the determinants of survival were gender, weight loss, performance status, PET/CT staging and multimodality therapy. Age was not a determinant. It would be expected that changing practice to not exclude treatment on age criteria alone could improve OS for the overall population.

Patients registered in the two tertiary centers had an increased likelihood of receiving comprehensive examination, staging work and active therapy. Through a detailed chart review for patients who had active therapy, it was noted there was a variation in practice between the two tertiary centers, with different utilization of invasive lymph node sampling and PET/CT noted. PET/CT scanning is known to influence management decisions and outcome in NSCLC [22-24]. The higher usage of PET/CT in tertiary centers could lead to stage migration. In addition, active treatment patterns varied between centers in this patient cohort.

The management of patients in clinical stage III NSCLC patients has more variability compared to patients with other stages of NSCLC [25] and this has been well described [2-3,5,8]. Evidenced-based treatment guidelines have been established in Alberta. We contend that the observed variations are explained by institutional characteristics such as a physician's individual judgment and personal beliefs around patient selection for treatment (Figure 2).

We acknowledge several limitations with our study. Firstly, the cancer registry did not provide information on performance status at initial visit, a factor known to influence physician decision-making around initial treatment [26]. Utilizing patients’ cancer center registration was a novel way to code for cancer service region of interest, but we contend a useful proxy for capturing the urban/rural divide. The study also uses ACRG scores as a proxy for performance status rather than the more commonly used Charlson index to measure comorbidity. An advantage is that it is readily calculated from inpatient and outpatient encounters, thereby permitting risk adjustment and overcoming limitations derived from both the Charlson and Elixhauser methods. In addition, ACRGs not only categorize individuals’ illnesses but include their severity and, as such, they present a unique opportunity to include individual patient factors.

## Conclusions

The organization of the cancer care system in Alberta has demonstrated a gap in how it provides access to treatment to meet the needs of its lung cancer patients, likely influencing survival outcomes. The government of Alberta has begun to address this issue in care by rolling out the Alberta Radiation Therapy Corridor. It will be of interest to patients and decision makers alike to examine how and to what degree the provision of radiotherapy and improved access to specialized caregivers in the community will now mitigate variations in cancer care and improve patient outcomes.
